# Editorial: Chemical composition, processing, and health-promoting potential of unconventional food plants

**DOI:** 10.3389/fnut.2024.1368629

**Published:** 2024-03-07

**Authors:** Maria Anete Santana Valente, Daniela da Silva Oliveira, Ângela Giovana Batista, Ceres Mattos Della Lucia, Leandro de Morais Cardoso

**Affiliations:** ^1^Departamento de Nutrição, campus Governador Valadares, Universidade Federal de Juiz de Fora, MG, Brazil; ^2^Departamento de Farmácia e Nutrição, campus Alegre, Universidade Federal do Espírito Santo, ES, Brazil; ^3^Departamento de Nutrição e Saúde, campus Viçosa, Universidade Federal de Viçosa, MG, Brazil

**Keywords:** nutritional value, chemical composition, bioactive compounds, nutrients, unconventional edible plants

The 2030 Agenda for Sustainable Development has among its goals the eradication of poverty, the end of hunger, and malnutrition. Certainly, the achievement of this objective involves diversifying the diet and encouraging the identification and consumption of accessible, nutritious, and bioactive compound-rich alternative food sources.

Currently, there is a very limited number of plant species used in human nutrition. There are still many unconventional food plants (UFP) unknown or underutilized by the general population due to their low popularity, lack of use, biodiversity loss, and traditional knowledge decline.

Unconventional edible plants include fruits, leaves, roots, seeds, stems, and/or flowers of non-domesticated species that grow spontaneously in nature. Many of these plants have been widely incorporated into human diets as a result of increasing research into new sustainable food sources with high nutritional value, in addition to bioactive compounds.

The use of UFP in diet can promote cultural diversification, especially in family farming, as they are part of the dietary tradition of many communities around the world. They also contribute to promoting food sovereignty since their cultivation and consumption can value and preserve dietary traditions, diversify food offerings, reduce dependence on conventional crops, strengthen food and nutritional security, and ensure the human right to adequate and healthy food.

This Research Topic aims to gather contributions addressing the main challenges and issues related to the chemical composition, processing, and health-promoting potential of unconventional edible plants. A total of five manuscripts were included, comprising four original research articles and one review. Some of these conducted chemical compound characterization and analyzed the nutritional, metabolite, and bioactive compound composition of UFP and their potential activities in human health. Studies on techniques for the better stability of metabolites of interest, actions in metabolic pathways, and clinical trials will be found, along with records of regional uses.

Saikia et al. determined the chemical composition and bioactive compounds of *Ipomea aquatica*, an unconventional wild edible plant found in various regions of Asia, Africa, and Australia. They observed an attractive nutritional and bioactive compound profile with potential human health benefits. The *I. aquatica* is a source of essential fatty acids, dietary fibers, and has a considerable mineral composition, especially Iron and Calcium. Its phenolic compounds have significant antioxidant potential, with possible protective action against DNA damage and tumor-initiating and -propagating cells. The extract of *I. aquatica* demonstrated inhibition of α-amylase and α-glucosidase enzyme activities, with possible effects on blood glucose control, making it a promising edible UFP.

Zhao et al. conducted metabolic and bioactivity analyses of different parts of *Duhaldea nervosa*, widely used as a medicine and food additive in China, aiming to explore and expand its medicinal value. The metabolic profile of flowers, roots, stems, and leaves of *D. nervosa* was determined, and 174 non-volatile compounds were identified. Among these, a large number showed significant antioxidant capacity, with high intensity in flowers and roots. An inhibitory activity of α-glucosidase was also observed in leaves and flowers of *D. nervosa*.

Tang et al. studied the effects of different processing methods on the quality, flavor, and nutritional potential of *Platostoma palustre*, using LC-MS and HS-GC-MS to evaluate the influences of processing on its metabolites and volatile substances. *P. palustre* is an important medicinal and edible plant in China and Southeast Asian countries. Thus, this research becomes a reference for establishing standardized processing methods and maintaining the quality stability of the plant.

Herz et al. evaluated and identified, through a clinical trial, the biological effects of benzyl isothiocyanate from the plant *Tropaelum majus* in modulating prostaglandin P2. Due to the high variability in the observed response in different individuals, no effects of the intervention with *T. maju*s were observed. In the long term, identifying phenotypes that respond differently to a clinical intervention can improve the provision of personalized nutrition or phytopharmaceuticals from this UFP.

Finally, Luo et al. evaluated the nutritional composition of *Lindera pulcherrima* leaves and documented the usage practices by the population living in the region where this plant could be found. Its nutritional and economic potential was highlighted, including in the production of different types of food products.

We hope that this Research Topic “*Chemical composition, processing, and health-promoting potential of unconventional food plants*” further expands interest in unconventional foods, such as plant, algae, insects, mushroom, and animal underutilized sources, highlighting their fundamental role in a diverse, sustainable, and healthy diet soon.

## Author contributions

MV: Writing—original draft. DO: Writing—review & editing. ÂB: Writing—review & editing. CD: Writing—review & editing. LC: Writing—original draft, Writing—review & editing.

